# Regulation of TNF-Related Apoptosis-Inducing Ligand Signaling by Glycosylation

**DOI:** 10.3390/ijms19030715

**Published:** 2018-03-02

**Authors:** Olivier Micheau

**Affiliations:** 1 INSERM, UMR1231, Laboratoire d’Excellence LipSTIC, F-21079 Dijon, France; olivier.micheau@inserm.fr; 2 UFR Sciences de Santé, University Bourgogne Franche-Comté, UBFC, F-21079 Dijon, France

**Keywords:** TRAIL, death-receptor, ligand, glycosylation, apoptosis, galectin, aggregation, trafficking, evolution, carbohydrate binding proteins, DR4, DR5, DcR1, DcR2, TRAIL-R1, TRAIL-R2, TRAIL-R3, TRAIL-R4, TNFRSF10A, TNFRSF10B, TNFRSF10C, TNFRSF10D

## Abstract

Tumor necrosis-factor related apoptosis-inducing ligand, also known as TRAIL or APO2L (Apo-2 ligand), is a cytokine of the TNF superfamily acknowledged for its ability to trigger selective apoptosis in tumor cells while being relatively safe towards normal cells. Its binding to its cognate agonist receptors, namely death receptor 4 (DR4) and/or DR5, can induce the formation of a membrane-bound macromolecular complex, coined DISC (death-signaling inducing complex), necessary and sufficient to engage the apoptotic machinery. At the very proximal level, TRAIL DISC formation and activation of apoptosis is regulated both by antagonist receptors and by glycosylation. Remarkably, though, despite the fact that all membrane-bound TRAIL receptors harbor putative glycosylation sites, only pro-apoptotic signaling through DR4 and DR5 has, so far, been found to be regulated by *N*- and *O*-glycosylation, respectively. Because putative *N*-glycosylation sequons and *O*-glycosylation sites are also found and conserved in all these receptors throughout all animal species (in which these receptors have been identified), glycosylation is likely to play a more prominent role than anticipated in regulating receptor/receptor interactions or trafficking, ultimately defining cell fate through TRAIL stimulation. This review aims to present and discuss these emerging concepts, the comprehension of which is likely to lead to innovative anticancer therapies.

## 1. Introduction

All ligands and receptors of the TNF superfamily, with the exception of TRAIL (TNF-Related apoptosis-inducing ligand or APO2L), harbor putative *N*- and/or *O*-linked glycosylation sites [1]. Yet, only a limited number of studies have investigated whether these post-translational modifications play a role in regulating their signal transduction capabilities. Protein glycosylation is a complex process involving hundreds of distinct genes. It is estimated that more than 50% of the human proteome is glycosylated [2]. It is probably the most common and ubiquitous post-translational protein modification, resulting in the covalent linkage of complex oligosaccharide chains to transmembrane or secreted proteins. The most abundantly occurring forms of carbohydrate modifications are linked to asparagine (Asn) [3] and serine (Ser) or threonine (Thr) amino acids [4].

These carbohydrate chains are not solely involved in protein folding control—i.e., ensuring proper synthesis of polypeptides prior to their addressing at the cell surface or secretion—but are also directly involved in the fine-tuning of membrane-bound receptor signaling capabilities. They could affect TRAIL receptors directly by changing the folding or flexibility of the cysteine-rich domain (CRD), similar to the recent findings on the ectodomain of the LDL-receptor-related protein 6 (LRP6), the *N*-glycosylation of which was found to be critical to its folding and aggregation potential [5]. Alternatively, depending on their location and quality, carbohydrate moieties could also serve as binding domains for lectins, thus allowing changes in transmembrane receptor trafficking or cell surface arrangement. Since most membrane-bound proteins harbor these post-translational modifications, carbohydrate chains may also allow unexpected glycoprotein/glycoprotein interactions, resulting in the positive or negative regulation of a given pathway and, in particular, signal transduction induced by TRAIL receptors.

## 2. Membrane Proximal TRAIL DISC Formation and Signaling Regulation

**Figure 1:**
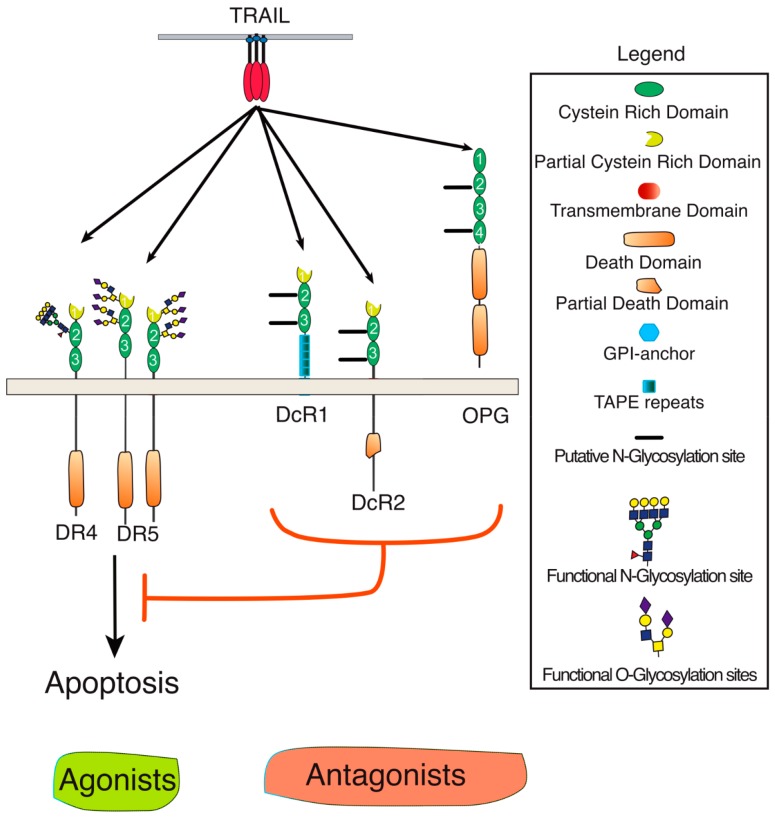
Schematic representation of TRAIL and its receptors. TRAIL and its agonist (DR4 and DR5) or antagonist (DcR1, Dcr2, or OPG) receptors are membrane-bound glycoproteins of the tumor necrosis factor (TNF) superfamily. DR stands for death receptor and DcR for decoy receptor. Specific domains or putative and described *O*- and *N*-glycosylation sites are depicted in the legend. Red T bar means inhibition.

**Figure 2:**
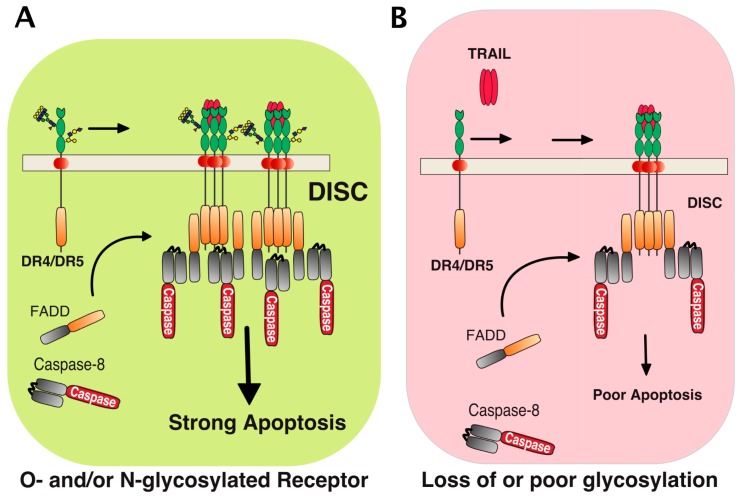
Schematic representation of TRAIL-induced death-inducing signaling complex DISC formation. TRAIL induced apoptosis in cancer cells is tightly associated with glycosylation of its agonist receptors, DR4 and DR5. (**A**) Stimulation of glycosylated DR4 or DR5 by TRAIL induces recruitment of the adaptor protein FADD and caspase-8, hence forming the so-called TRAIL DISC (or death-inducing signaling complex), where processing of the caspase-8 occurs. This allows the triggering of apoptosis. Carbohydrate transferases, including *N*-acetylgalactosaminyl-, fucosyl-, or sialyl-transferases, as well as galectins could act directly at the receptor level to regulate TRAIL DISC formation and activation (see text for details). (**B**) In cells displaying poor polypeptide *N*-acetylgalactosaminyltransferase activity [42] or expressing a non-glycosylable receptor [43], binding of TRAIL to the receptors is not altered, but DISC formation and processing of caspase-8 are restrained. This impairs TRAIL’s ability to trigger apoptosis.

TRAIL belongs to the TNF ligand superfamily. This family is composed of 19 ligands, each capable of binding to at least one of the 29 receptors described so far [1]. TRAIL is unique for its ability to bind with high affinity to four distinct receptors, namely DR4 (TRAIL-R1), DR5 (TRAIL-R2), DcR1 (TRAIL-R3), and DcR2 (TRAIL-R4). It can also bind with the soluble receptor osteoprotegerin (OPG), albeit with much lower affinity (Figure 1) [6,7]. Because signal transduction induced by TRAIL has been associated with the induction of apoptosis in tumor cells early on, the targeting of this cytokine or its receptors has prompted major interest in oncology [8,9]. TRAIL-induced apoptosis is solely induced through DR4 and DR5, owing to the presence of the death domain (DD) within their intracellular domain. DD is a homotypic protein interaction domain [10]. The DD is necessary and sufficient for the recruitment of the adaptor protein FADD which, in turn, enables the association of the initiator caspases [11], including caspase-8 (Figure 2). Recruitment of these initiator caspases to DR4 and/or DR5 leads to their activation within the so-called DISC (death-inducing signaling complex). Once activated, initiator caspases are released into the cytosol, allowing for the cleavage and activation of effector caspases, such as caspase-3, which act directly on specific cellular substrates to dismantle the cells by apoptosis [12]. DcR1 and DcR2 are unable to transduce apoptosis upon TRAIL binding due to the lack of a functional death domain (Figure 1). Like OPG (osteoprotegerin) (which is a secreted soluble receptor) and despite the fact that OPG harbors two DD, these receptors have long been considered as decoy receptors [13,14]. While most of these antagonist receptors behave as decoy receptors when co-expressed with agonist receptors on the cell surface or within the tumor microenvironment [15], DcR2 has, in addition, been found to be able to interact directly with both DR4 and DR5, restraining caspase-8 recruitment and activation [16,17]. DcR2 has also been found to be capable of transducing signaling pathways such as NF-κB or AKT (Protein kinase B) [18,19,20].

As inferred from studies on Fas/CD95, another member of the TNF family which also harbors a DD, caspase-8 activation within the TRAIL DISC is thought to occur by proximity [21,22]. Quantitative mass spectrometric studies suggest that recruitment and activation of caspase-8 within the DISC occurs via the formation of caspase-8 chains [23,24]. Irrespective of the stoichiometry, the requirement for a high oligomerization order for proper caspase-8 activation implies that a specific arrangement of the receptor is required to allow efficient apoptosis triggering. Consistent with this model, forced aggregation of DD-containing receptors of the TNF superfamily is often sufficient to increase caspase-8 activation [25,26], whereas, on the other hand, co-recruitment of DcR2 within the TRAIL DISC decreases caspase-8 recruitment and arrangement. This, thus, prevents its activation and apoptosis induced by TRAIL [17,27,28].

Within the TRAIL DISC, other proteins are also co-recruited with the caspase-8 [12,29], including, but not restricted to c-FLIP. This is probably the most important intracellular inhibitor caspase involved in the initiation of the extrinsic pathway [27,30,31,32]. However, unlike glycosylation or TRAIL antagonist receptors, downstream DISC components are not specific to TRAIL signaling. Most of them are also recruited to and regulate signal transduction induced by other receptors of the TNF superfamily [33,34,35,36,37,38,39] and beyond. An example of other such receptors are the toll-like receptors, including TLR3 [40,41].

## 3. Agonist TRAIL Receptors are Glycosylated

**Figure 3:**
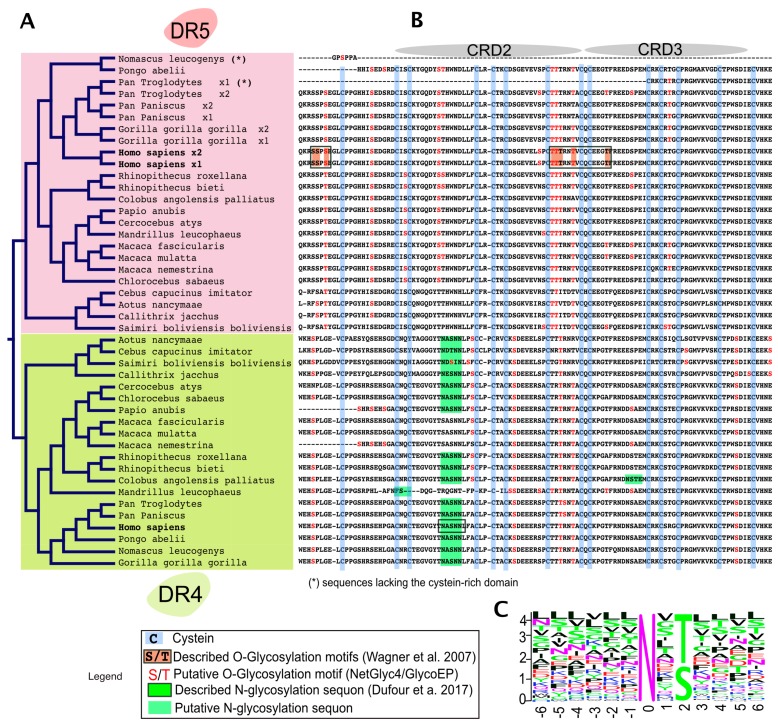
Alignment comparison of the cysteine-rich domains of DR4 and DR5 in primates. (**A**) Dendrogram of DR4 and DR5 full primate sequences; (**B**) a corresponding Clustal Omega multiple sequence alignment showing the region encompassing the two-cysteine rich domains of DR4 and DR5 [66]. The legend illustrates how cysteine residues and putative or described *O*- or *N*-glycans are depicted in the alignment; (**C**) sequence logo displaying residues preferentially placed at occupied *N*-glycan sequons, adapted from Weng et al. [67]. Neighboring residues located downstream (positions +6) and upstream (positions −6) from the asparagine residue (position 0) are also shown. The height of each letter represents the residue prevalence or frequency at the putative position.

Proper arrangement of the TRAIL DISC scaffold has been found to be controlled by glycosylation. In 2007, the first demonstration showing that *O*-linked glycosylation contributes to DR5 pro-apoptotic potential was published (Figure 2 and [42]). Using a whole genome-profiling approach in a large collection of tumor cell lines, the authors found that cell sensitivity to TRAIL-induced cell death was tightly associated with elevated levels of polypeptide *N*-acetylgalactosaminyltransferases, such as GALNT14. Consistent with this observation, it was found that inhibition of this *O*-glycosyltransferase using siRNAs impairs TRAIL-induced cell death (Table 1). DR5 was demonstrated in this study to be *O*-glycosylated on two stretches of serines and threonines within or surrounding the CRDs 2 and 3 (Figure 3). Mutagenesis of these serines and threonines to alanine prevented *O*-glycosylation of DR5 and limited its ability to transduce apoptosis [42]. Importantly, it was also found that TRAIL binding affinity to the receptor was neither increased nor decreased whether DR5 was *O*-glycosylated or not. Mechanistically, albeit it remains unclear how glycosylation precisely regulates apoptosis induced by TRAIL, the authors could show that the sugar moieties could directly affect caspase-8 recruitment and activation at the level of the TRAIL DISC, indicating that these carbohydrate chains are likely to play a direct role in the fine structuration of the DISC and/or the devenir of the membrane-bound complex. It should be emphasized here that, in contrast to DR5, physiological and biological evidence for DR4 *O*-glycosylation remains poor in this study, raising the question of whether *O*-glycosylation also accounts for efficient signal transduction of apoptosis through DR4.

Sequence alignment of the extracellular domains, encompassing CRD2 and CRD3, of DR4 and DR5 in primates, indicates that both receptors share amino acid sequence homology with respect to serines and threonines as well as predicted putative *O*-glycosylation sites (Figure 3). Strikingly, however, while DR5 primate sequences lack putative *N*-glycosylation sites, DR4 sequences (with the exception of Macacas) contain at least one sequon, suggesting that *N*-glycosylation may occur and regulate signal transduction through this receptor.

**Figure 4:**
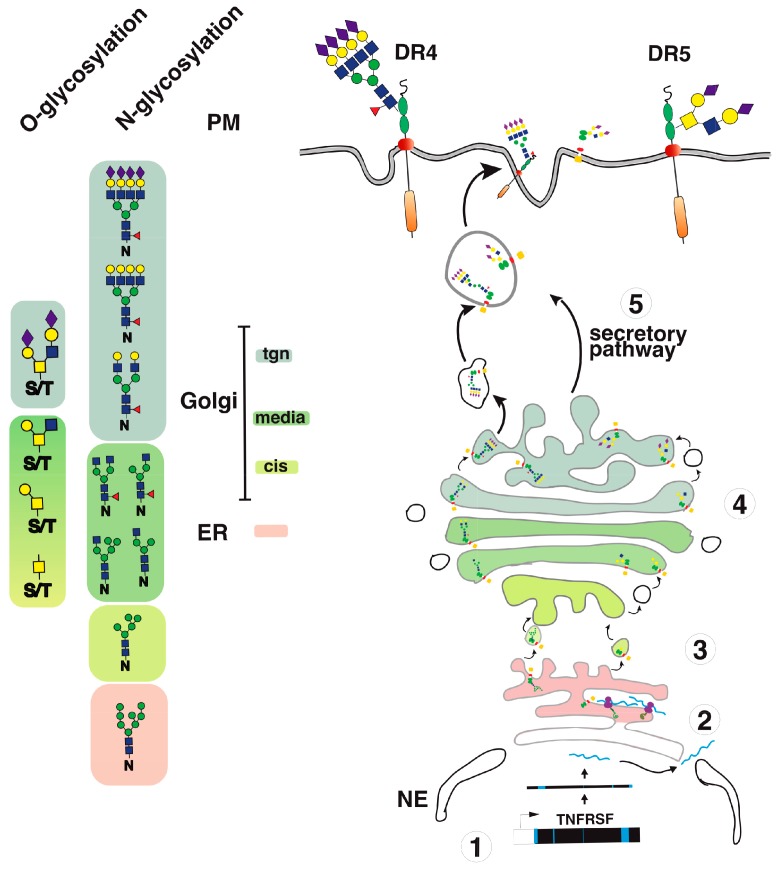
TRAIL receptor glycosylation and trafficking. A simplified illustration of DR4 and DR5 glycosylation and trafficking to the cell surface. (**1**) Once transcribed; (**2**) upon translation; (**3**–**4**) TRAIL receptors undergo glycosylation from the ER to the Golgi apparatus. (**5**) This post-translational modification occurs stepwise in the Golgi apparatus, allowing glycan maturation and complexification prior to cell surface transport via vesicular trafficking.

Consistent with a regulatory function associated with the *N*-glycosylation of these receptors, DR4 was found to be *N*-glycosylated in human tumor cell lines [44] and this translational modification was demonstrated to be required for apoptosis induced by this receptor ([43] and Figure 2). This carbohydrate modification also occurs within the CRD2 on an asparagine located in position 156 of human DR4 (Figure 3). Like DR5, TRAIL binding affinity for DR4 was found to remain unchanged, irrespective of whether the receptor is glycosylated or not [43]. Mechanistically, it was found that receptor aggregation, initiator caspase recruitment and activation within the DISC, and apoptosis were increased when DR4 was *N*-glycosylated. These events were reduced or impaired in human cancer cells expressing a non-glycosylable form of DR4 [43]. These findings are in sharp contrast to previous findings demonstrating that the inhibition of *N*-glycosylation [45,46,47,48,49], endoplasmic reticulum (ER) and Golgi stressors [50,51,52,53], or glucose deprivation [54,55,56,57] increase tumor cell sensitivity to TRAIL-induced cell death (Table 1). Keeping in mind that these compounds are either pan-inhibitors of *N*-glycosylation and of the unfolded protein response (UPR) pathway or competitive inhibitors of glucose metabolism—such as the tunicamycin, thapsigargin or 2-deoxyglucose (2DG)—conclusions drawn from these experiments may not apply to a given transmembrane protein such as DR4. Indeed, these inhibitors are not specific to TRAIL agonist receptors and, albeit some of them might coincidentally induce cell death via DR4 and/or DR5 (Table 1), they often induce drastic changes of glycosylation in all proteins expressed by the targeted cells or induce the collapse of the Golgi apparatus and/or the ER. Mechanistically, these compounds have been found to induce the upregulation of DR5, as well as to decrease the expression levels of important inhibitors such as c-FLIP [52,54,56], IAPs [48], or Bcl-2 inhibitors [51,55]. Some of them also induce, alone, a ligand-independent cell death program that involves DR4 and DR5 [58,59,60,61]. By perturbing or inducing the collapse of the ER or the Golgi apparatus, these drugs also alter the glycosylation of transmembrane receptors. In particular, they alter *O*- or *N*-linked glycosylation of receptors such as DR4 and DR5, but not only these (Figure 4). It thus appears relatively clear that only gene editing studies followed by rescue experiments with point mutations of the putative glycosylated sites, such as that published by Dufour et al. [43], can address the importance of these carbohydrate chains in regulating TRAIL-induced cell death.

## 4. TRAIL Receptor Glycosylation during Evolution

**Figure 5:**
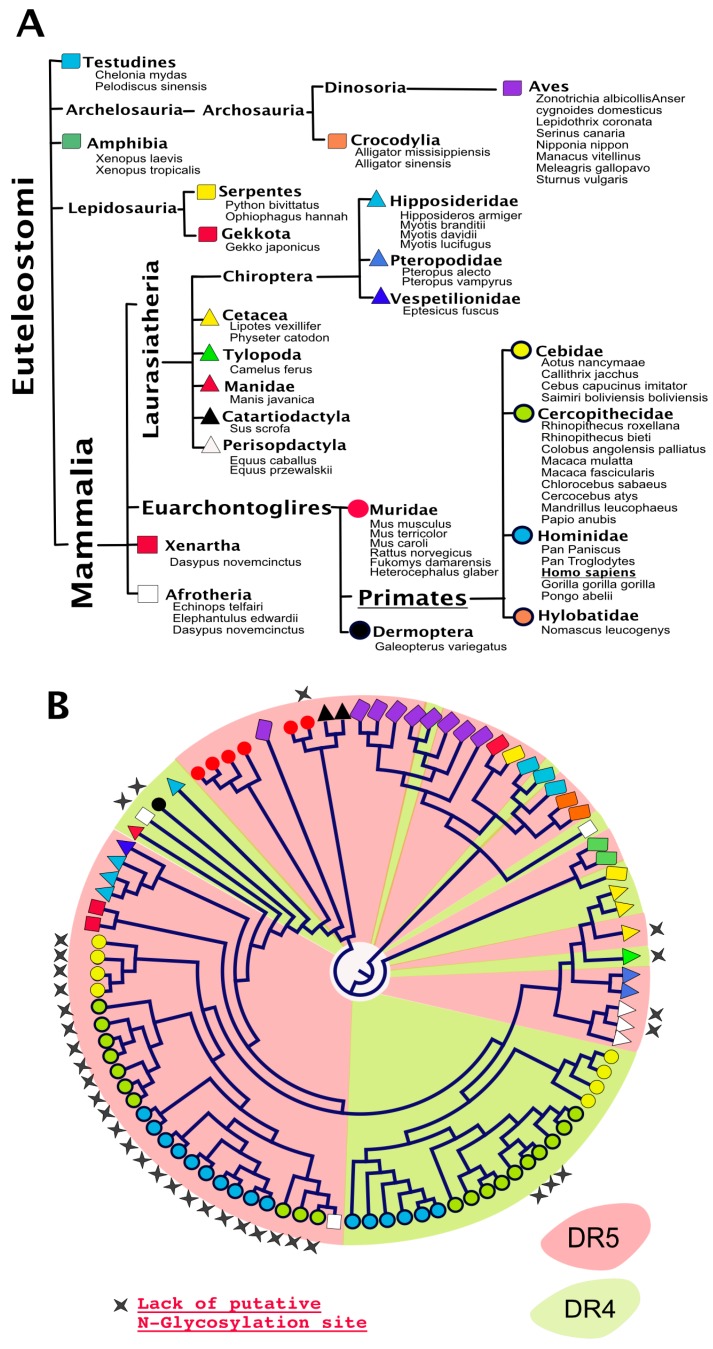
Phylogeny of DR4 and/or DR5 in “bony vertebrates”. (**A**) DR4 and DR5 are, so far, exclusively reported in the Euteleostomi clade. Orders and families in which DR4 and DR5 are expressed are depicted here using distinctly colored symbols; (**B**) phylogenetic distribution of DR4 (green) and DR5 (red). Stars above the circular cladogram point to sequences devoid of putative *N*-glycosylation sites.

**Figure 6:**
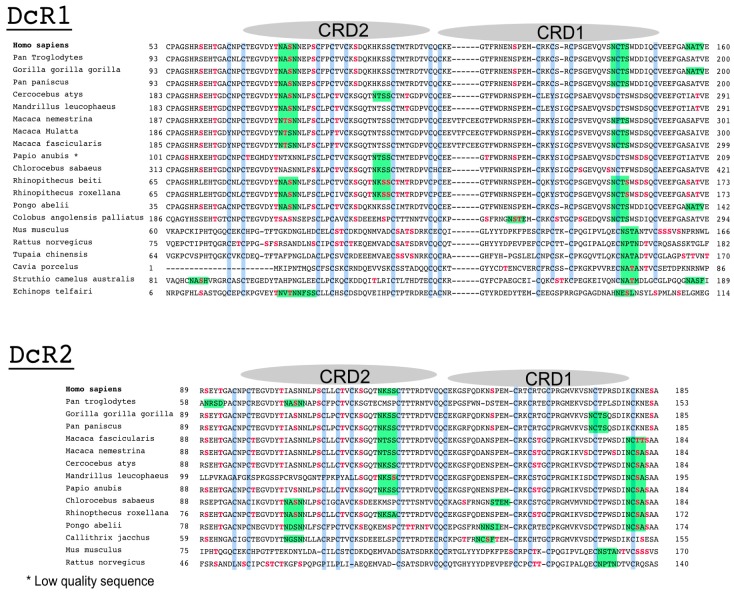
Alignment comparison of the cysteine-rich domains of DcR1 and DcR2. Corresponding Clustal Omega multiple sequence alignment showing the region encompassing the two cysteine-rich domains of DcR1 and DcR2 in different species [66].

Analysis of putative *N*-glycosylation sites in orthologue DR4 and/or DR5 sequences reveal that, with a few exceptions, almost all TRAIL agonist receptors harbor putative *N*-glycosylation sites (Figure 5). Moreover, it is also worth noting that the presence of conserved putative *N*-glycosylation sites extends beyond agonist receptors—since such sequons are also found in TRAIL antagonist receptors—within the extracellular region encompassing CRD1 and CRD2 (Figure 6). Like many other genes found in higher organisms, DR4 and DR5 arise from a gene duplication during evolution. DR4 and DR5 are the two flavors of an original gene which remains present as a single version in lower organisms such as rodents (see also Figure 5). Interestingly enough, with the exception of DR5 primate sequences and a few others, most TRAIL agonist receptor sequences harbor at least one *N*-glycosylation site, including the unique TRAIL agonist receptor expressed in lower organisms which is often defined as the orthologue of DR5. It would thus be tempting to speculate that *N*-glycosylation represents the most ancient form of carbohydrate modification linked to the original TRAIL agonist receptor and beyond the TNFR superfamily. Because this feature has been preserved during evolution, it was not surprising to find that *N*-glycosylation plays also a major regulatory function with regards to TRAIL receptor/receptor interactions and signal transduction in lower organisms. Accordingly, the unique mouse TRAIL agonist receptor has also been demonstrated to be *N*-glycosylated on three asparagines located in positions 99, 122, and 150. This corresponds to CRD2 and CRD3 [43]. Similar to DR4, *N*-glycosylation of mTRAIL-R was found to increase apoptosis induced by TRAIL, without inhibiting or enhancing TRAIL binding to its receptor [43]. Rather, like DR4 and DR5, the glycosylation of mTRAIL-R was associated with better receptor aggregation at the cell surface.

## 5. TRAIL Receptor Clustering

**Figure 7:**
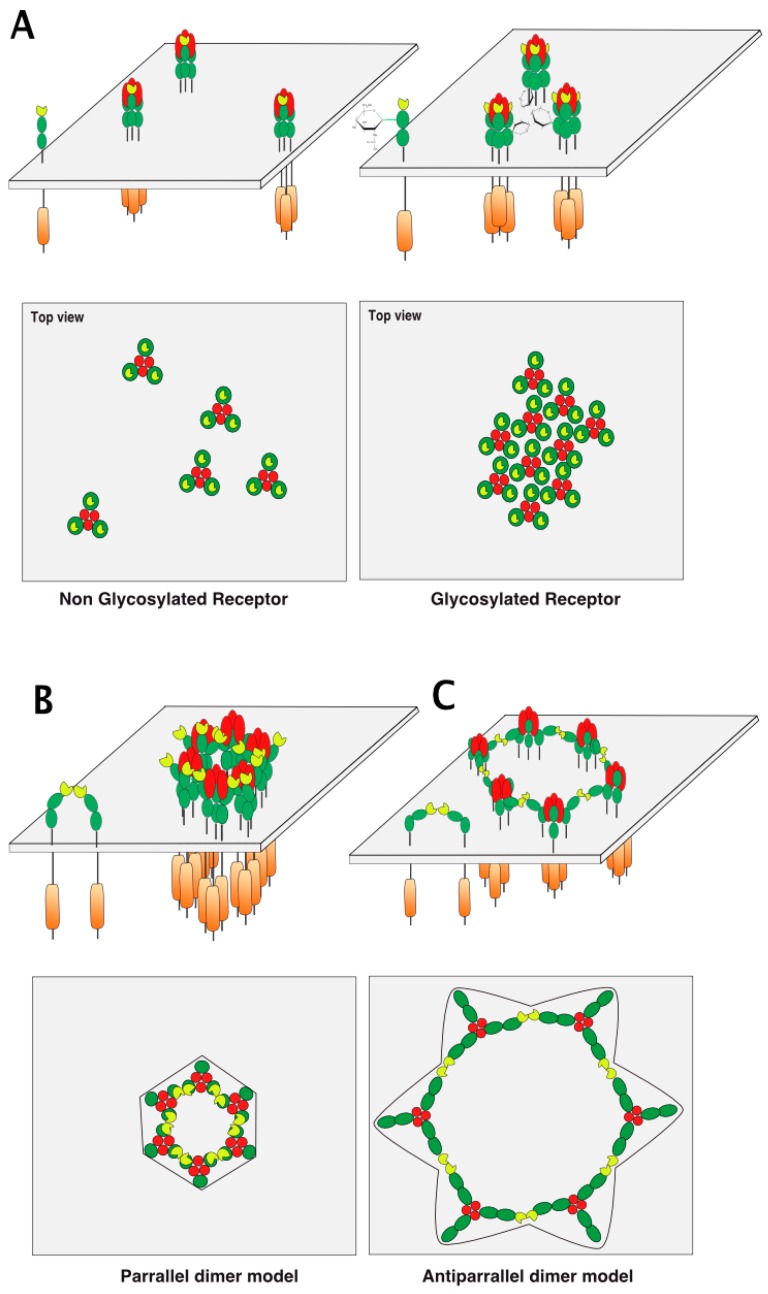
TRAIL receptor aggregation models. (**A**) Illustration of the basic ligand/receptor organization consisting of a trimeric ligand binding three distinct receptors. The carton below is a hypothetical representation of the clustering of DR4 or mTRAIL-R according to respective glycosylation status viewed from the top of the cell membrane; (**B**) PLAD-based parallel and (**C**) antiparallel dimer models adapted from Vanamee et al. [71].

How glycosylation favors receptor clustering or helps proper formation of the TRAIL DISC scaffold remains unclear (Figure 7A). Several models of receptor cluster/aggregation formation have been proposed so far, based on structure/function studies of TNF/TNF receptors [68,69,70], as well as crystallographic studies of TRAIL or agonist antibodies binding to TRAIL receptors [71]. While ligands of the TNF superfamily are known to preassemble spontaneously as trimers [72], TNF receptors, albeit long thought to be monomeric, are able to form dimers on the cell surface [68,73]. Spontaneous assembly of TNF receptor homo- or hetero-dimers was found to be mediated by the pre-ligand assembly domain or PLAD [16,39,70], a stretch of amino acids residing within the partial CRD1 (Figure 1). The PLAD is particularly interesting with respect to TRAIL receptors, since this dimeric binding-domain has been demonstrated to allow heteromeric interactions between DR5 and DcR2 in the absence of TRAIL [74]. It should be noted, however, that this interaction is not only maintained but also increased in the presence of TRAIL, as demonstrated experimentally by native TRAIL DISC immunoprecipitation [17]. This implies that models describing receptor arrangement through dimeric interactions are likely to apply to heteromers too.

Hexameric arrangement models, based on parallel or antiparallel dimers of TRAIL receptors have been proposed, explaining how receptor clustering leads to efficient apoptotic signal transduction (Figure 7B,C). These models are inferred from crystallographic analysis of the ternary complex formed by the anti-DR5 (AMG655) Fab fragment, TRAIL, and DR5 [75] and mutational analysis of DR5 [76]. It should be kept in mind that activation of caspase-8 occurs through proximity [22], irrespective of whether the caspase-8 is able to form chains within the DISC [23,24]. Therefore, arrangement of TRAIL receptors and, thus, caspase-8 vicinity within the antiparallel model is likely to be less favorable for efficient caspase-8 activation than the parallel model, which supports closer caspase-8/caspase-8 interactions (Figure 7).

Whether glycosylation directly affects these arrangements remains to be determined. However, it may be possible to envision the possibility that carbohydrate chains allow the binding of lectins to regulate receptor lattice formation.

## 6. TRAIL Signal Transduction Regulation by Carbohydrate-Binding or Modifying Proteins

**Figure 8:**
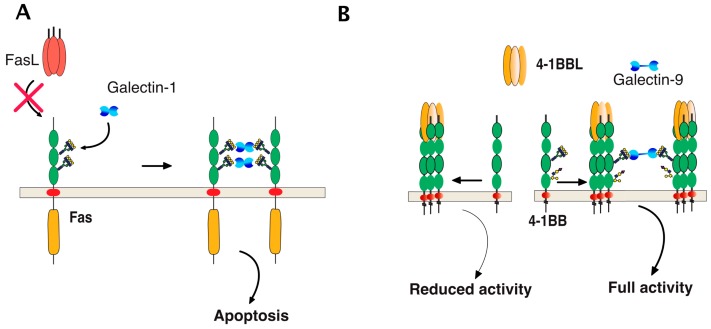
Schematic representation of galectin-mediated Fas and 4-1BB clusterin, two receptors of the TNF receptor superfamily. (**A**) Galectin-1 induced apoptosis can occur through its binding with Fas in a ligand-independent manner due to the fact that Fas is *N*-glycosylated [77]. The red cross illustrates the lack of requirement of FasL for this interaction to occur; (**B**) binding of 4-1BBL to its cognate receptor 4-1BB, a glycoprotein harboring *N*-glycosylation sites promoting CD4 and CD8 T-cell activation. Full activation of 4-1BB has recently been found to require direct binding of galectin-9 to 4-1BB [81,82].

**Figure 9:**
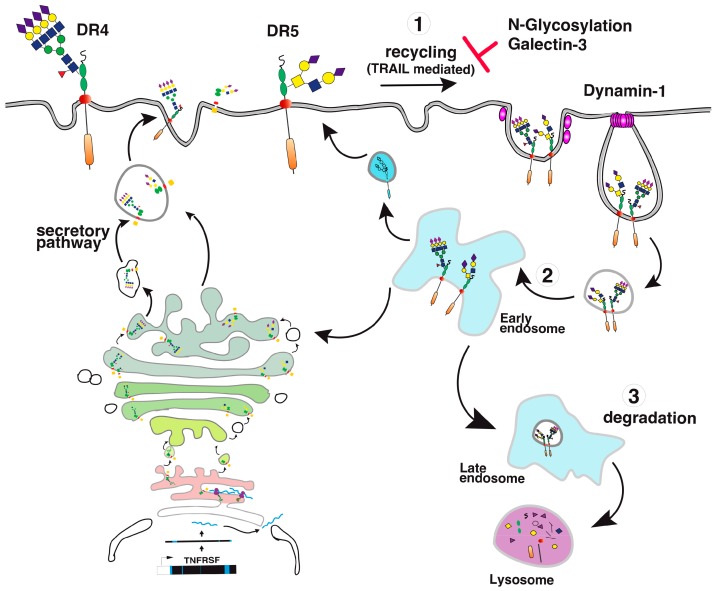
TRAIL receptor internalization and recycling after TRAIL stimulation. A simplified illustration of DR4 and DR5 internalization. (**1**) After TRAIL binding, the receptor aggregates and undergoes fast internalization in a clathrin/dynamin-1 dependent manner. Receptor internalization has been found to be negatively regulated by galectin-3 [83] and restrained by glycosylation [43]. (**2**) In early endosomes, the receptors can then be recycled to the cell surface (**3**) or routed to late endosomes for degradation.

Direct binding of galectins to glycosylated TNF receptors has seldom been studied. However, increasing evidence suggests that these interactions are likely to be more frequent and important than expected. It has been found, for example, that galectin-1 could induce apoptosis in two leukemic T cell lines, namely Jurkat and CEM, owing to its ability to bind directly to Fas [77]. Since Fas is *N*-glycosylated [78,79], the binding of galectin-1 to Fas in these cells is thought to be sufficient to induce Fas clustering, caspase-8 recruitment, and activation of the initiator caspase in the absence of its cognate ligand (Figure 8). More recently, 4-1BB, another receptor of the TNF superfamily which is also *N*-glycosylated [80], has been demonstrated to interact with and to require galectin-9 (Figure 8) for efficient signal transduction [81,82]. Regulation of TRAIL-induced signaling by galectins has also been described. However, direct interactions of β-galactoside-binding proteins with TRAIL receptors have not been described to date, with the exception of a publication in 2012 describing the inhibition of TRAIL receptors endocytosis by galectin-3 [83]. In this work, Mazurek et al. could demonstrate that a colon cancer cell line, stably generated for TRAIL-resistance after repetitive exposition to TRAIL, gained insensitivity to TRAIL-induced apoptosis by elevating cell surface expression of galectin-3 (Figure 9). Elevation of galectin-3 expression was associated with reduced TRAIL receptor endocytosis and a resistance to TRAIL. Inhibition of galectin-3 using shRNAs or selective inhibitors restored TRAIL sensitivity and receptor internalization [83]. Moreover, inhibition of DR4 and DR5 endocytosis after TRAIL stimulation was directly associated with galectin-3 cell surface expression and physical interaction between TRAIL receptors and galectin-3. This was demonstrated by the immunoprecipitation of the receptors and immunoblotting or by immunofluorescence [83]. Galectins are devoid of peptide signal and transmembrane domain, but they can be found both in the cytosol or on the cell surface after secretion. Despite it remaining unclear how the latter are being secreted, their retention on the cell surface has recently been found to require *N*-glycosylated plasma membrane receptors or lipids [84], which would support the finding that galectin-3 could act directly at the cell surface to regulate apoptosis induced by TRAIL. The findings of Mazurek et al. do not rule out the possibility that the accumulation of galectin-3, which is associated with a reduction of TRAIL receptor endocytosis after TRAIL stimulation in their clone, may be driven by an alteration of the clathrin-dynamin-1 endocytic pathway [85] (Figure 9). It is worth mentioning here that a number of studies have been produced describing the pro-apoptotic properties of galectins [86,87,88,89] or their ability to interfere with cell death signal transduction, including apoptosis induced by ligands of the TNF superfamily ([83,90,91,92,93,94,95] and Table 2). One of them has provided evidence, for instance, that galectin-3 can bind to the intracellular domain of Fas [96] and account for the so-called type I or type II cell subtypes which define cells according to their requirement to induce apoptosis through an amplification loop induced by mitochondria [97]. In this study, it was found that galectin-3 would transform type II cells to type I and, therefore, allow complete apoptosis in a mitochondria-independent manner [96]. Studies focused on TRAIL have mostly reported indirect modulation of the extrinsic pathway by galectins. Likewise, in the reconstitution of galectin-3 in the TRAIL-resistant breast cancer cell line BT459, expressing low levels of the lectin was found to restore apoptosis induced by TRAIL through a mechanism associated with a decrease in AKT phosphorylation [90]. It was next demonstrated that a restoration of TRAIL-induced cell death by galectin-3 in BT459 cells required its phosphorylation on serine-6 [98] and that TRAIL sensitivity may be influenced by a sequence polymorphism commonly found in galectin-3 and associated with breast cancer incidence [94]. However, in contrast, in the human bladder carcinoma cell line J82 or in papillary thyroid cancer cells, overexpression of galectin-3 was found to impair cell death induced by TRAIL through an increase of phosphorylation of Akt on serine 473 [91,93]. Lastly, it has also been described that galectin-1 could impair TRAIL-induced apoptosis in human hepatocellular carcinoma cells, a finding associated with a change in survivin and Bcl-2 expression [95]. Unlike the studies demonstrating functional regulation of 4-1BB signal transduction by galectin-9 [81,82], none of the studies mentioned above has addressed whether the galectins, due to their potential ability to bind to glycosylated TRAIL receptors, are likely to specifically regulate apoptosis by interfering or enhancing receptor aggregation.

**Figure 10:**
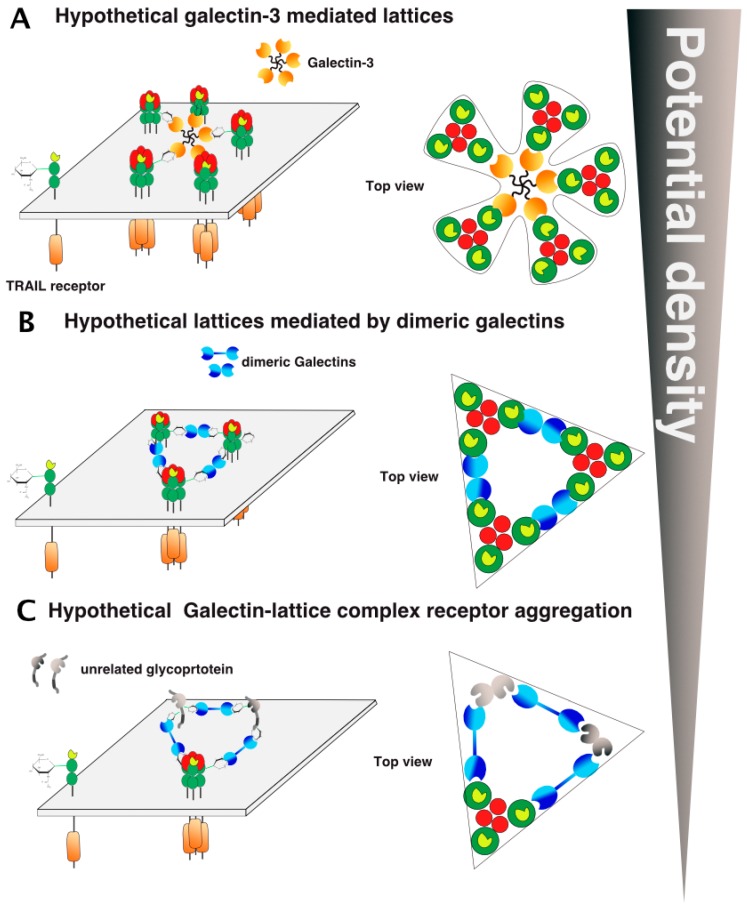
Galectin/TRAIL receptor hypothetical aggregation models. Several models involving galectins could explain how glycosylation regulates TRAIL DISC formation and activation, including carbohydrate-mediated interactions with galectins such as lattice formation. (**A**) An illustration of the hypothetic galectin-glycan-receptor lattice organization is shown with the pentameric galectin-3 and (**B**) dimeric galectins. (**C**) Inclusion of non-TRAIL receptor-related glycoproteins to TRAIL receptor clusters, gathered by galectins. Potential density here stands for cell membrane density of the receptors.

However, keeping in mind that the arrangement and clustering of TRAIL receptors at the cell surface after TRAIL stimulation represents a crucial step to induce efficient caspase-8 activation, proteins displaying affinity with carbohydrates, such as galectins, are likely to play a crucial regulatory role in the process. Similar to the PLAD dimeric models (Figure 7B,C), galectins, owing to their affinity for β-galactosides, are likely to interact directly with DR4 or DR5 and to contribute to their spatial organization within the plasma membrane. As illustrated in Figure 10, at least two hypothetical models that may also enrich or exclude the PLAD dimeric models could explain how galectins may enhance or inhibit TRAIL signaling. This can be done merely by reducing the distance between TRAIL/receptor-based units or, in contrast, by increasing the distance between trimerized receptors (Figure 10). As illustrated in this figure, depending on their valency, galectins are likely to differentially shape TRAIL receptor arrangement on the cell surface, either increasing or inhibiting signal transduction. Sugar moieties may be involved or required for heteromeric TRAIL receptor formation. Such a scenario could apply to TRAIL inhibitory receptors, as these also harbor putative *N*-glycosylation sites (Figure 6).

Alternatively, since all TRAIL receptors are glycosylated and are thus potential binding-partners for galectins, other arrangements may be proposed, including unexpected interactions with non-TNF receptor-related glycoproteins (Figure 10). In the figure, these interactions have been illustrated as inhibitory, but the latter could, contrarily, increase TRAIL receptor aggregation and density on the cell surface or stabilize the TRAIL DISC, thus allowing proper caspase-8 activation and apoptosis triggering.

**Figure 11:**
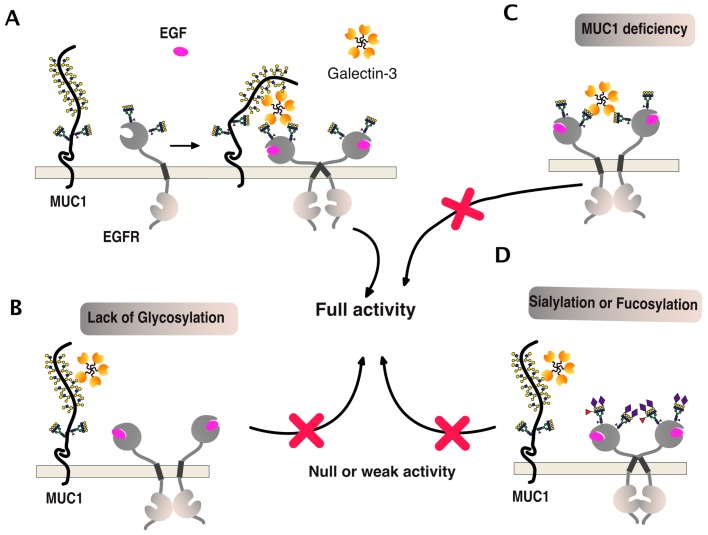
EGFR multimerization and signaling, illustrating how carbohydrate-binding proteins or carbohydrate-transferases can directly affect transmembrane receptor signal transduction. (**A**) To be optimal, activation of EGFR signaling was found to involve both a galectin, here being galectin-3, and the glycoprotein MUC1 [100,106]. (**B**–**D**) Illustrations depict how the qualitative carbohydrate decoration of EGFR glycosylation or MUC1 deficiency can impair EGFR signaling. Red crosses indicate that activation of this signaling pathway is compromised, as compared to the situation presented in panel A.

One of the best examples of such a positive association can be illustrated by the epithelial growth factor receptor (EGFR) signaling (Figure 11). This receptor is both *O*- and *N*-glycosylated and, like DR5, its signal transduction activity has been described to be tightly associated with polypeptide *N*-acetylgalactosaminyltransferases [99] and galectins [100,101]. As illustrated in Figure 11, EGFR carbohydrate sugar chains support complex ternary interactions between the receptor itself, galectin-3, and MUC1. A deficiency of any of these partners or changes in glycosylation, such as sialylation of fucosylation, can disrupt the fine organization of the scaffold, thus leading to reduced EGFR dimerization and signal transduction [100,102,103,104,105,106,107,108].

On this topic, it is worth noting that glycan-modifying enzymes can also alter tumor necrosis factor receptor superfamily (TNFRSF) members’ signal transduction capabilities, including cell death induced by DD-containing receptors [109]. Likewise, it was found, early on, that Fas could be sialylated in B and T cell lymphoma cell lines. Mere removal of sialic acids in these cell lines using a neuraminidase was found to be sufficient to restore sensitivity to Fas-induced cell death [110,111]. Because the carbohydrate binding affinity of galectins is usually compromised by sialylation [112], these findings would support the hypothesis that galectins may positively contribute to Fas ligand-induced cell death. Consistent with this possibility, it has been demonstrated that the glycosyltransferase, ST6Gal-I (which adds sialic acid in α-2,6 to *N*-glycans), induces Fas sialylation and inhibits apoptosis-induced by this receptor [113]. Intriguingly, however, although the authors of this study suggested that sialylation would not affect TRAIL signaling, the results presented in this manuscript suggest that sialylation may also prevent TRAIL-induced cell death, at least to some extent. It shall be kept in mind, though, that ST6Gal-I would only be effective on DR4 which is *N*-glycosylated [43] but not on DR5 which is *O*-glycosylated [42]. This differential glycosylation status could, therefore, explain why the effects of sialylation were less pronounced towards TRAIL-induced apoptosis than Fas ligand in this study. This is because DR5, which is unlikely to be affected by ST6Gal-I, would remain fully capable of transducing apoptosis upon TRAIL stimulation. In agreement with this hypothesis, TNFR1 (which is also subject to *N*-glycosylation [114]) has also been found to be modified by ST6Gal-I and, similar to Fas, its signal transduction efficacy was also found to be impaired by sialylation [115].

Conversely, fucosylation, another oligosaccharide modification, may contribute to apoptosis induced by TRAIL [42,70,71]. A deficiency in GDP-mannose-4,6-dehydratase (GMDS), a GDP-mannose converting enzymes essential for de novo fucosylation in colorectal cancer cells, was found to confer resistance to TRAIL [70]. Of interest, the authors of this study demonstrated that fucosylation of DR4, but not DR5, restored TRAIL sensitivity in these cells [71].

## 7. Conclusions

DD-containing receptors of the TNF super family have been known, since the beginning, for their propensity to self-aggregate and to trigger apoptosis, even in the absence of their cognate ligand [10,116]. TRAIL agonist receptors also display this tendency [117,118]. Investigators have been searching a long time for an explanation for the lack of self-receptor aggregation at the cell surface, explaining why cells harboring the latter fail to undergo spontaneous apoptosis in the absence of the ligand. In the late 1990s, a protein coined silencer of death domain (SODD) or silencer of death domain was proposed to bind the DD-containing receptor TNFR1, thus avoiding recruitment and activation of the caspase-8 [116]. It turned-out, however, that SODD knock-out mice were viable and born at the expected Mendelian ratio, and that cells deficient for SODD would not undergo spontaneous apoptosis nor display differential sensitivity to TNF-induced apoptosis [119]. Given that a growing body of evidence indicate that glycosylation is likely to contribute to the regulation of TNF receptor family signaling and aggregation, it could be interesting to determine whether and how these post-translational modifications control receptor self- or ligand-induced aggregation. Keeping in mind that sialylation and fucosylation, depending on the lactosamine modification [120], can differentially impair galectin binding to carbohydrate chains, the results gathered so far (albeit apparently discrepant) may be unified into a more general model explaining how glycosylation plays a prominent role in the fine-tuning of these potentially harmful receptors. Future studies will be required to determine whether this interplay offers the cell the ability to control receptor aggregation, cell surface arrangement, trafficking, and apoptosis. Increasing our understanding of these complex interactions could additionally open unexpected therapeutic options valuable in oncology and in autoimmune diseases.

## Figures and Tables

**Table 1:** Enzymes and inhibitors involved or regulating protein glycosylation found to alter TRAIL-induced cell death (see text for details). ⬆ stands for an increase whereas ⬇ indicates a decrease in protein expression or complex formation.

Target/Compound	Cell Type	TRAIL-Induced Apoptosis	Comment	Reference
Carbohydrate-binding proteins				
Tunicamycin	Colon carcinomas	Enhanced	⬆ DR5 and ⬇ EGFR	[49]
	Colon and Lung carcinomas	Enhanced	UPR-mediated ⬆ DR5	[58]
ER/Golgi Stressor	Multiple Myeloma			
	Oral cancer cells	Enhanced	⬆ DR5	[62]
2-deoxy-d-glucose	Melanomas	Enhanced	⬆ DR5	[63]
	Melanomas	Enhanced	⬆ DR5	[57]
Glucose deprivation	Leukemias, Breast and Cervical carcinomas	Enhanced	⬆ TRAIL DISC formation and ⬇ of c-FLIP	[54,60]
	Colon carcinomas	Enhanced	ATF4-mediated ⬆ of DR4 and DR5	[61]
Fucosylation (GDMS)	Colon carcinomas	Inhibited		[64,65]
benzyl-a-GalNAc	Pancreatic cancer Melanoma Colon carcinomas	Inhibited	⬆ TRAIL DISC formation and ⬆ receptor aggregation	
siGalnt14		Inhibited		
siGalnt3		Inhibited		[42]
siFUT6		Inhibited		
GALNT14 overexp.		Enhanced		

**Table 2:** Carbohydrate-binding proteins found to alter TRAIL-induced cell death (see text for details). ⬆ stands for an increase whereas ⬇ indicates a decrease in protein expression.

Target/Compound	Cell Type	TRAIL-Induced Apoptosis (*FasL)	Comment	Reference
Carbohydrate-binding proteins				
siGal1	Hepatocellular Carcinomas	Enhanced	⬇ Survivin and ⬇ Bcl-2	[95]
Gal3 P64→H64	Breast Carcinomas	Enhanced		[94]
siGal3	Papillary Thyroid Carcinomas	Enhanced	⬇ AKT	[93]
phospho-Gal3	Breast Carcinomas	Enhanced	⬇ AKT	[98]
Gal-3 Overexp.	Bladder Carcinomas	Inhibited	⬆ AKT	[91]
	Breast Carcinomas	Enhanced	⬇ AKT	[90]
	Leukemias	Enhanced	converts type II cells into type I	[96]
Gal3 extracellular	Colon carcinomas	Inhibited	Inhibition of receptor trafficking (membrane level)	[83]

## Abbreviations

AKT	protein kinase B
Asn	asparagine
c-FLIP	cellular FLICE (FADD-like IL-1β-converting enzyme)-inhibitory protein
CRD	cysteine-rich domain
DcR	decoy receptor
DcR1	decoy receptor 1, TRAIL-R3, TNFRSF10C
DcR2	decoy receptor 2, TRAIL-R4, TNFRSF10D
DD	death domain
DISC	death-inducing signaling complex
DR	death receptor
DR4	death receptor 4, TRAIL-R1, TNFRSF10A
DR5	death receptor 5, TRAIL-R2, TNFRSF10B
EGFR	epithelial growth factor receptor
ER	endoplasmic reticulum
Fas	fibroblast associated surface antigen, also known as CD95
FADD	Fas associated death domain
Fas-L	Fas ligand
NE	nuclear envelope
NF-κB	nuclear factor-kappa B
OPG	osteoprotegerin
PLAD	pre-ligand assembly domain
PM	plasma membrane
Ser	serine
SODD	silencer of death domain
shRNA	short hairpin RNA
siRNA	small interfering RNA
Thr	threonine
TNF	tumor necrosis factor
TNFR1	tumor necrosis factor receptor 1
TRAIL	TNF related apoptosis inducing ligand, also known as APO2L
